# Supplementary material for: PD-L1 Expression Is Significantly Associated with Tumor Mutation Burden and Microsatellite Instability Score
Source: Cancers (Basel). 2021 Sep 16;13(18):4659. doi: 10.3390/cancers13184659 (PMC8466224; doi:10.3390/cancers13184659)
Supplement: Supplementary file 1 [file cancers-13-04659-s001.zip › cancers-1339232-supplementary.pdf]

cancers MDPI

Supplementary

PD-L1 Expression Is Significantly Associated with Tumor Mutation Burden and Microsatellite Instability Score

Yoon Ah Cho, Hyunwoo Lee, Deok Geun Kim, Hyunjin Kim, Sang Yun Ha, Yoon-La Choi, Kee-Taek Jang and Kyoung-Mee Kim

Table S1. Calculation of Tumor mutational burden (TMB). TMB = (Total eligible variants (N))/(Total coding region ≥50X coverage (N))

Trusight Oncology 500 and Whole-exome Sequencing	
**Eligible Variants**	**Non-eligible Variants**
SNVs and indels	
In coding regions	In non-coding regions
In high-confidence regions*	In low-confidence regions
With ≥ 50× coverage	With high COSMIC counts (≥50)
With ≥ 5% VAF	Multinucleotide variants
	Filtered germline variants
	Found in germline variant databases†
	Additional variants flagged based on VAF

Abbreviations: single nucleotide variant, SNV; insertion and deletion, indel; variant allele frequency, VAF; * High-confidence regions defined as more than 50x coverage. † Germline variant databases include gnomAD, ExAC, and dbSNP.

Table S2. Overall sequencing quality of 310 cases.

	Average ± 2SD	Recommended Guideline Quality Threshold
Median insert size	110.11 ± 8.61	≥70
Median exon coverage	592.81 ± 286.56	≥150
Percentage of exon 50× (%)	98.74 ± 3.71	≥90.0
Usable MSI sites	98.14 ± 25.03	≥40
Total passing filter reads	88714647.10 ± 12462333.05	≥80,000,000
Mean family size (duplication)	2.621613 ± 1.716591	N/A
Percentage of target 0.4× mean	83.54 ± 8.91	N/A

Abbreviations: MSI, Microsatellite instability; N/A, not available.

Table S3. Tumor mutation values of TSO500 and Whole exome sequencing.

Case No.	Coding Region Size of TSO 500 (MBp)	Number of Eligible Variants of TSO 500	TMB of TSO 500	Interpretation of TSO 500	Coding Region Size of WES (MBp)	Number of Eligible Variants of TSO 500	TMB of WES	Interpretation of WES
Case 1	1.28	110	85.9	TMB-High	35.9	1469	40.9	TMB-High
Case 2	1.28	9	7	TMB-Low	35.9	84	2.3	TMB-Low
Case 3	1.28	12	9.4	TMB-Low	35.9	147	4.1	TMB-Low
Case 4	1.28	122	95.4	TMB-High	35.9	2182	60.8	TMB-High
Case 5	1.28	87	68	TMB-High	35.9	1012	28.2	TMB-High
Case 6	1.28	6	4.7	TMB-Low	35.9	74	2.1	TMB-Low
Case 7	1.28	4	3.1	TMB-Low	35.9	68	1.9	TMB-Low

TMB, Tumor mutation burden; TSO 500, Trusight oncology 500; WES, whole-exome sequencing.

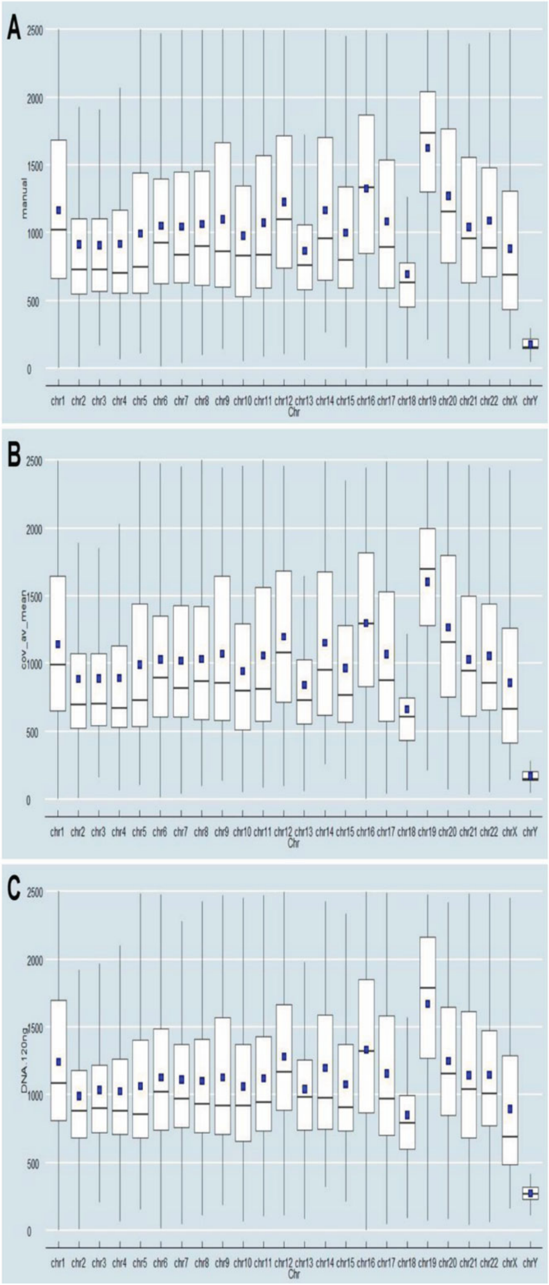

Figure S1. The average depth of coverage according to DNA extraction method and input. (**A**) Average depth of coverage using the beads pooling method. (**B**) Average depth according to the manual method (**C**) Increase in the average depth of coverage due to increase in the DNA input from 80 ng to 120 ng.

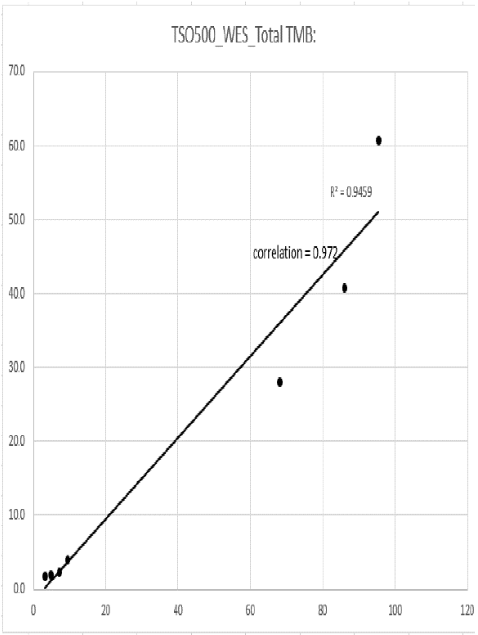

Figure S2. Comparison measurement of tumor mutation burden (TMB) between TruSight oncology 500 (TSO 500) and whole-exome sequencing (WES). They showed high concordance between the TMB measurements from the TSO 500 and WES analyses. TMB values from both assays exhibited a high concordance (R^2 = 0.972).
